# *Lupinus angustifolius* Protein Hydrolysates Reduce Abdominal Adiposity and Ameliorate Metabolic Associated Fatty Liver Disease (MAFLD) in Western Diet Fed-ApoE^−/−^ Mice

**DOI:** 10.3390/antiox10081222

**Published:** 2021-07-29

**Authors:** Guillermo Santos-Sánchez, Ivan Cruz-Chamorro, Ana Isabel Álvarez-Ríos, José María Fernández-Santos, María Victoria Vázquez-Román, Beatriz Rodríguez-Ortiz, Nuria Álvarez-Sánchez, Ana Isabel Álvarez-López, María del Carmen Millán-Linares, Francisco Millán, Justo Pedroche, María Soledad Fernández-Pachón, Patricia Judith Lardone, Juan Miguel Guerrero, Ignacio Bejarano, Antonio Carrillo-Vico

**Affiliations:** 1 Instituto de Biomedicina de Sevilla, IBiS (Universidad de Sevilla, HUVR, Junta de Andalucía, CSIC), 41013 Seville, Spain; gsantos-ibis@us.es (G.S.-S.); beatriz.rodriguezortiz@unican.es (B.R.-O.); nalvarez-ibis@us.es (N.Á.-S.); aialvarez-ibis@us.es (A.I.Á.-L.); plardone@us.es (P.J.L.); guerrero@us.es (J.M.G.); ibejarano@us.es (I.B.); 2 Departamento de Bioquímica Médica y Biología Molecular e Inmunología, Universidad de Sevilla, 41009 Seville, Spain; 3 Departamento de Bioquímica Clínica, Unidad de Gestión de Laboratorios, Hospital Universitario Virgen del Rocío, 41013 Seville, Spain; anai.alvarez.sspa@juntadeandalucia.es; 4 Departamento Citología e Histología Normal y Patológica, Universidad de Sevilla, 41009 Seville, Spain; jmsantos@us.es (J.M.F.-S.); mvazquez2@us.es (M.V.V.-R.); 5 Cell Biology Unit, Instituto de la Grasa, CSIC, Ctra, Utrera Km 1, 41013 Seville, Spain; mcmillan@ig.csic.es; 6 Department of Food & Health, Instituto de la Grasa, CSIC, Ctra, Utrera Km 1, 41013 Seville, Spain; fmillanr@ig.csic.es (F.M.); j.pedroche@csic.es (J.P.); 7 Área de Nutrición y Bromatología, Departamento de Biología Molecular e Ingeniería Bioquímica, Universidad Pablo de Olavide, Ctra Utrera Km 1, 41013 Seville, Spain; msferpac@upo.es

**Keywords:** lupin, bioactive peptides, NAFLD, oxidative stress, inflammation, adipose tissue, steatosis, cholesterol, LDL

## Abstract

Metabolic-associated fatty liver disease (MAFLD) is the most important cause of liver disease worldwide. It is characterized by the accumulation of fat in the liver and is closely associated with abdominal obesity. In addition, oxidative stress and inflammation are significant features involved in MAFLD. Recently, our group demonstrated that lupin protein hydrolysates (LPHs) had lipid lowering, antioxidant, and anti-inflammatory effects. Sixty male mice fed with a Western diet were intragastrically treated with LPHs (or vehicle) for 12 weeks. Liver and adipose tissue lipid accumulation and hepatic inflammatory and oxidant status were evaluated. A significant decrease in steatosis was observed in LPHs-treated mice, which presented a decreased gene expression of CD36 and LDL-R, crucial markers in MAFLD. In addition, LPHs increased the hepatic total antioxidant capacity and reduced the hepatic inflammatory status. Moreover, LPHs-treated mice showed a significant reduction in abdominal adiposity. This is the first study to show that the supplementation with LPHs markedly ameliorates the generation of the steatotic liver caused by the intake of a Western diet and reduces abdominal obesity in ApoE^−/−^ mice. Future clinical trials should shed light on the effects of LPHs on MAFLD.

## 1. Introduction

Metabolic-associated fatty liver disease (MAFLD) is the principal cause of chronic liver disease worldwide, with an estimated global prevalence of 25–30% [1,2]. MAFLD is associated with metabolic risk factors such as insulin resistance, hyperlipidemia and central obesity [3]. In fact, MAFLD prevalence can range 67–91% in obese subjects, depending on the BMI and waist circumference [4]. MAFLD presents a wide spectrum of clinical phenotypes that range from simple steatosis (SS) to non-alcoholic steatohepatitis (NASH) [5]. SS is characterized by the presence of more than 5% lipids in hepatocytes while NASH is characterized by advanced steatosis, ballooning, and lobular inflammation [6]. Frequently, NASH may progress to fibrosis, hepatic cirrhosis, and finally to hepatocellular carcinoma, which can end up with the need for a liver transplant [5].

Regarding the pathophysiology of MAFLD, the theory of “multiple hits model” has replaced the earlier “two hits hypothesis” in which obesity and insulin resistance generate an hepatic accumulation of lipids (first hit) that activate oxidative stress and an inflammatory cascade and fibrogenesis (second hit) [7]. The multiple hits model maintains the formers hits (oxidative stress and inflammation), but adds a more complex vision of human MAFLD, including genetic and dietary factors, interactions with other organs, and metabolic dysfunction [8]. Thus, several studies have demonstrated a dysregulation of triglycerides [9] and cholesterol [10] metabolism in MAFLD which is associated with the severity of the disease. This dysregulation affects receptors, hormones, and transcription factors, causing lipid accumulation in the liver [11]. In fact, an increase in the expression of hepatic enzymes involved in triglycerides synthesis, such as fatty acid synthase (FASN) [12], and the cluster of differentiation (CD) 36 [13], which promote the long-chain fatty acids uptaking in the liver, has been shown in MAFLD patients. In contrast, the low density lipoprotein receptor (LDL-R) expression, implicated in the cholesterol metabolism, is reduced [10].

Furthermore, MAFLD is strongly associated with the endocrine cross-talk between liver and adipose tissue [14]. In this regard, adiponectin receptor 2 (AdipoR2), the liver receptor of adiponectin, a protein secreted from adipose tissue that has been shown to possess anti-inflammatory, anti-lipidemics, and anti-diabetic effects [15], has been suggested to play an important role in the pathogenesis of MAFLD [15].

The diet have also been suggested to contribute to MAFLD development [16]. In this regard, diets rich in saturated fats and added sugar contribute to the hepatic accumulation of fats, to increase the oxidative stress and the inflammatory response [8,17,18]. Moreover, several studies have demonstrated that healthy changes in dietary habits can promote a clinically meaningful regression of disease status [19]. For this reason, life-style interventions based on nutritional changes is considered the first line therapy in preventing or ameliorating MAFLD [20]. In this way, plant peptide such as those from soy have been shown not only to lower the MAFLD status in animals [21] and humans [18] but also to be able to decrease the main obesity risk factors [21].

Apolipoprotein E (ApoE) plays a pivotal role in fat metabolism and lipid homeostasis by binding to LDL-R and ApoE-specific remnant receptors [22]. Previous studies have demonstrated that ApoE absence predisposes to hypercholesterolemia and atherosclerosis [23,24]. Besides, ApoE Knockout mice (ApoE^−/−^) have a higher inflammatory and oxidative stress status compared to wild type mice [25]. Moreover, a seven weeks treatment of Western Diet (WD) has been shown to be enough to induce MAFLD in ApoE^−/−^ mice [25,26] unlike other models that need longer induction time. Therefore, the use of WD in ApoE mice reduces time and cost. In addition, previous reports have shown that WD, besides increase hyperlipidemia, exacerbates inflammation and oxidative stress promoting metabolic syndrome and MAFLD generation with great similarity to the human disease [26].

Our group has recently demonstrated that lupin protein hydrolysates (LPHs), obtained from hydrolysis of *Lupinus angustifolius*, exert blood lipid-lowering, antioxidant, and anti-inflammatory effects [27,28]. Given that growing evidence emphasizes that changes in the diet cause correction of obesity and the subsequent remission of steatosis [19,29], we hypothesize that LPHs treatment could reduce the risk in developing MAFLD. Therefore, the aim of this study was to evaluate the effect of LPHs on abdominal obesity and liver status of ApoE^−/−^ mice fed a WD.

## 2. Materials and Methods

### 2.1. LPHs Preparation

LPHs were obtained at the Instituto de la Grasa of Seville (Consejo Superior de Investigaciones Científicas, Seville, Spain) as described previously [27].

### 2.2. Animals, Experimental Protocol and Dosage Information

**Figure 1:**
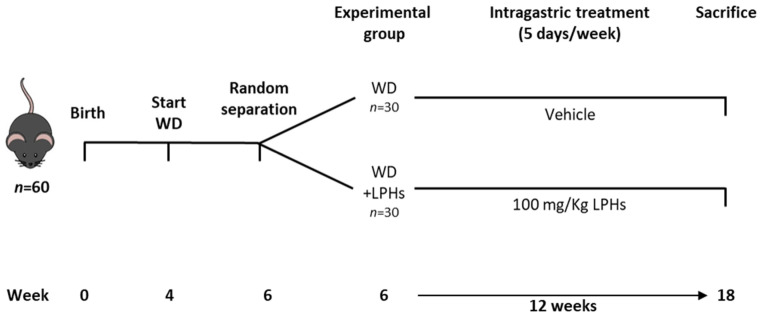
Experimental design and timeline. Schematic diagram of the experimental design of the study. WD, Western diet; LPHs, lupin protein hydrolysates.

The experimental design is shown in Figure 1. Sixty 4-week-old male ApoE^−/−^ mice were housed in the animal facility of Instituto de Biomedicina de Sevilla (IBiS) under standard conditions (12/12 light/dark cycles, temperature 22 ± 2 °C and humidity <55%) with free access to water and WD (Test Diet 58v8, 45% Energy from Fat). The composition of WD is shown in supporting information Table S1. After two weeks, mice were randomized into two groups and intragastrically treated five days/week with vehicle (WD group) or LPHs (WD+LPHs group) for 12 weeks. LPHs were administered at 100 mg/Kg dissolved in 0.9% saline and 0.25% carboxymethyl cellulose (CMC) (Sigma Aldrich, St. Louis, MO, USA). WD mice received the same treatment without LPHs. LPHs dose was selected based on previous internal tests. The human equivalent dose was 8.12 mg/Kg calculated, according to [30]. Daily food intake and individual body weight were measured weekly and recorded. At the experiment endpoint (18 weeks), fasted animals were euthanized with an intraperitoneal injection of sodium thiopental (50 mg/Kg, B. Braun Medical SA, Barcelona, Spain) and the blood was removed by perfusion with phosphate-buffered saline (PBS) for 5 min through a Thermo FH100 peristaltic pump (Thermo Fisher Scientific, Vantaa, Finland). Abdominal white adipose tissue (WAT) and liver were subsequently collected, weighed and stored until used. The experimental protocol was performed under the Spanish legislation and the EU Directive 2010/63/EU for animal experiments and was approved by the Virgen Macarena and Virgen del Rocío University Hospitals ethical committee (reference 21/06/2016/105). Initial ApoE^−/−^ mice to generate the colony were kindly gifted by Dr. Antonio Ordoñez and Dr. Raquel del Toro (IBiS).

### 2.3. Liver Lipids Quantification

Liver was homogenized in chloroform:isopropanol:Nonidet P-40 (Sigma Aldrich) (7:11:0.1 *v*/*v*/*v*). Samples were centrifuged at 10,000× *g* for 10 min at 4 °C and the supernatant was collected and dried in a Jouan RC1022 evaporator (Thermo Fisher Scientific). The pellet obtained was dissolved in saline 1% Nonidet P-40. Hepatic total cholesterol (hTC) and hepatic triglycerides (hTG) levels were measured through a chemiluminescence immunoassay using the COBAS E 601^®^ modular analyser (Roche Diagnostics, Basel, Switzerland).

### 2.4. Histological Analysis

Frozen liver samples were sectioned at 10 μm and postfixed in 40% formaldehyde. Slides were then stained with Oil Red O (Sigma Aldrich) for 10 min, counterstained with Harris’s hematoxylin (PanReac AppliChem, Chicago, IL, USA) containing 4% acetic acid for 2 min, and finally cover-mounted for observation. Paraformaldehyde-fixed liver and WAT samples were routinely dehydrated, paraffin-embedded, and sectioned at 2–4 μm. Slides were then stained with hematoxylin-eosin (PanReac AppliChem, Barcelona, Spain) and cover-mounted for observation. Photomicrographs were obtained using an Olympus photomicroscope (Vanox AHBT3, Olympus, Tokyo, Japan) and Nikon (DS-Fi310, Nikon, Tokyo, Japan) digital camera. Adipocyte morphology (area and size) and hepatic steatosis degree (lipid-droplet total area, number per cell, and average area) were estimated using the NIH ImageJ v1.52p free software package (NIH, Bethesda, MD, USA). Data were collected from an average of 200 cells, two hematoxylin-eosin-stained sections per mouse. Histopathological changes were analysed blindly by two independent experimenters.

### 2.5. Antioxidant Capacity

The ferric reducing antioxidant power (FRAP) assay was measured in liver homogenate according to Escudero-López et al. [31]. 280 µL of FRAP reagent were mixed with 20 µL sample. After 30 min of incubation at 37 °C, the absorbance (595 nm) was read with a Synergy™ HT-multimode microplate reader (Biotek Instruments, Winooski, VT, USA). The results were obtained by data extrapolation with a calibration curve using 6-hydroxy-2,5,7,8-tetramethylchroman-2-carboxylic acid (Trolox, Sigma Aldrich). Lipid peroxidation was measured through the quantification of malondialdehyde (MDA) and 4-hydroxynonenal (4-HNE). 40 µL of the sample was mixed with 130 µL methanol:acetonitrile (1:3 *v*/*v*) solution, that contained the chromogen *N*-methyl-2-phenylindole. Then, 30 µL methanesulfonic acid were added, incubated for 15 min at 60 °C and incubated for 5 min on ice to stop the reaction. Moreover, the activity of the antioxidant enzymes CAT, GPx (Cayman Chemical, Ann Arbor, MI, USA), SOD (Arbor Assays, Ann Arbor, MI USA) and GR (Biovision, Milpitas, CA, USA) were measured in liver homogenate according to the manufacturer’s indications. The absorbances were measured with a CLARIOstar Plus microplate reader (BMG Labtech, Ortenberg, Germany). Liver homogenate was carried out as follow. 100 mg frozen liver were homogenated in 4 mL of PBS containing protease inhibitor cocktail (Sigma Aldrich) according to the manufacturer’s instructions using the TissueRuptor II (Qiagen, Hilden, Germany). Samples were centrifuged at 12,000× *g* for 15 min at 4 °C and the supernatant was collected and frozen until use. Protein content was measured according to Lowry protein assay by using the DC (detergent compatible) protein assay (Bio-Rad, Hercules, CA, USA).

### 2.6. RNA Isolation and RT-qPCR

Livers were homogenized in TriSure Isolation Reagent (Bioline, Luckenwalde, Germany) using the TissueRuptor II. RNA isolation was carried out according to the manufacturer’s instructions. cDNA was obtained by using the Transcriptor First Strand cDNA Synthesis Kit (Roche). Quantitative PCR was performed with 80 ng cDNA/well in the Lightcycler 480 thermocycler with the LightCycler 480 SYBR Green I Master kit (all from Roche). Hypoxanthine phosphoribosyltransferase (*hprt*) was used as the housekeeping gene and the relative expression levels were calculated using the 2^−ΔΔCt^ method. Primers used: *AdipoR2,* forward (Fwd) 5′-ATTTGGAGCCCAGCTTAGAGA-3′, reverse (Rev) 5′-GCCTTCCCACACCTTACA-3′ (NM_197985), annealing temperature (Ta) = 54 °C; *CD36*, Fwd 5′-ATTCATTTGTTCAAGTTGTGC-3′, Rev 5′-AAAAGGTGGAAAGGAGGCTGC-3′ (NM_001159558), Ta = 52 °C; *Fasn*, Fwd 5′-AGAGATCCCGAGACGCTTCTG-3′, Rev 5′-GCCTGGTAGGCATTCTGTAGT-3′ (NM_007988), Ta = 56 °C; *Ldl-r,* Fwd 5′-TCAGACGAACAAGGCTGTCC-3′, Rev 5′-CCATCTAGGCAATCTCGGTCTC-3′ (NM_010700), Ta = 61 °C; *Sr-a*, Fwd 5′-GGCTGGAGGGAAGTTGTCAATAC-3′, Rev 5′-TCTGCTGCATCCCACTGGTGA-3′ (NM_025291), Ta = 54 °C; *Hprt*, Fwd 5′-TGTTGGATATGCCCTTGACTA-3′, Rev 5′-TGCGCTCATCTTAGGCT-3′ (NM_013556), Ta = 52.61 °C.

### 2.7. Statistical Analysis

The statistical analysis was performed using SPSS^®^ statistic software v25 (IBM Corporation, Armonk, NY, USA). Data were expressed as the mean and standard error of the mean (SEM). Data did not follow a normal distribution so the differences between the two experimental groups were evaluated through the unpaired and non-parametric Mann-Whitney U test. Correlations were analysed by the non-parametric Spearman’s correlation. Values of *p* ≤ 0.05 were considered statistically significant.

## 3. Results

### 3.1. LPHs Treatment Does Not Change Body Weight and Calorie Intake

As shown in Supporting Information Figure S1, no differences were observed either in the baseline weights (BBW) or in the final body weight (FBW) in WD+LPHs-treated mice compared to WD group. Furthermore, no changes in the body weight gain (BWG) and the daily food intake (DFI) were observed between groups.

### 3.2. LPHs Ingestion Reduces Abdominal WAT Size

**Figure 2:**
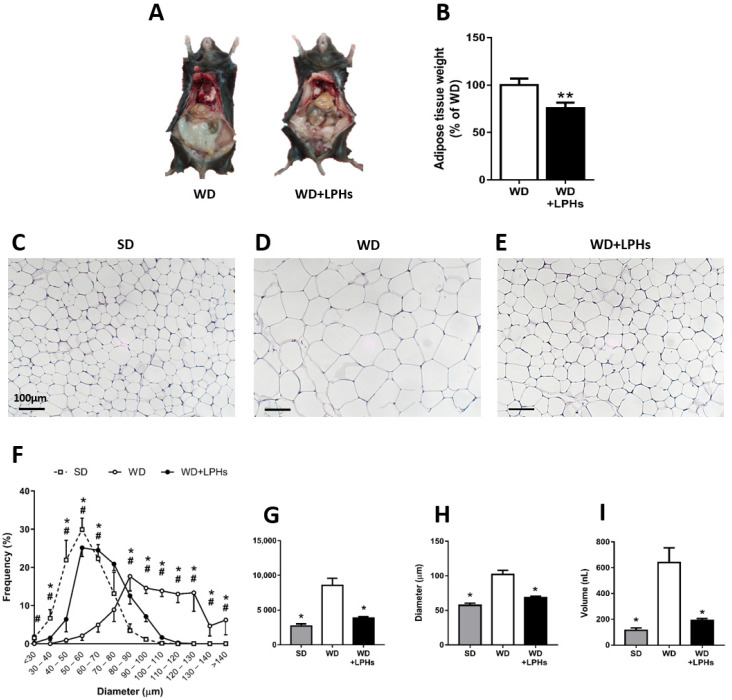
White adipose tissue analysis. Representative image of the abdominal WAT (**A**). White adipose tissue quantification (**B**) (*n* = 30). Representative images of hematoxylin/eosin staining of WAT from SD (**C**), WD (**D**), and WD+LPHs (**E**) groups. Frequency of mean adipocyte size (**F**), mean adipocyte area (**G**), diameter (**H**), and volume (**I**). Values are shown as the mean and the standard error of the mean of each group. * *p* ≤ 0.05 respect to the WD group. ** *p* ≤ 0.01 respect to the WD group. In (**F**), # *p* ≤ 0.05 between SD and WD, * *p* ≤ 0.05 between WD and WD+LPHs. SD, standard diet group; WD, western diet group; WD+LPHs, western diet + lupin protein hydrolysates group.

After 12 weeks of LPHs-treatment, a significant reduction in the abdominal WAT was observed in comparison with the WD group (Figure 2A,B). Also, the histological analysis of WAT sections showed that the number of adipocytes was significantly higher in the WD group than WD+LPHs-treated mice (Figure 2C–E). A lower frequency of very large adipocytes (>80 µm) was observed in WD+LPHs-treated mice in comparison with the WD group (Figure 2F). In addition, WD+LPHs group showed a significant decrease in adipose hypertrophy, by the reduction in area (Figure 2G), diameter (Figure 2H), and volume (Figure 2I) of adipocytes. In fact, WD+LPHs mice showed a similar frequency of large adipocytes and mean area, diameter and volume per adipocyte (Figure 2F–I) to the standard diet (SD) group.

### 3.3. LPHs Decrease the Hepatic Steatosis

**Figure 3:**
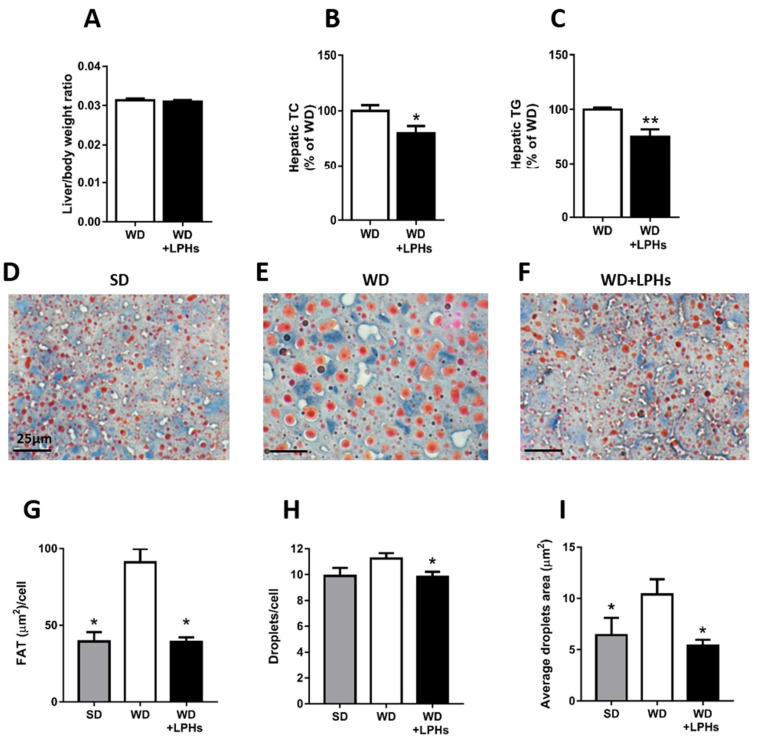
Hepatic lipid profile. Liver/body weight ratio (**A**) (*n* = 30). hTC (**B**) and hTG (**C**) content of both experimental groups (*n* = 15). Representative images of Oil red O staining of liver tissue from SD (**D**), WD (**E**), and WD+LPHs (**F**) groups. Quantitative analysis of fat area (**G**), droplets/cell (**H**), and average droplets area (**I**). Values are shown as the mean and the standard error of the mean of each group. * *p* ≤ 0.05 with respect to the WD group. ** *p* ≤ 0.01 with respect to the WD group. SD, standard diet group; TC, total cholesterol; TG, triglycerides; WD, western diet group; WD+LPHs, western diet + lupin protein hydrolysates group.

hTC and hTG concentrations were significantly reduced in WD+LPHs-treated mice in comparison with the WD group without affecting liver/body weight ratio (Figure 3A–C) To confirm the reduction of LPHs-induced steatosis, oil red staining was performed in the liver. LPHs-treated mice showed a significant reduction of hepatic lipid accumulation compared to the WD group (Figure 3D–J). Interestingly, the lipid accumulation observed in the WD+LPHs group was similar to the fat concentration present in the liver of SD-fed mice. Fat area (Figure 3G), fat droplets/cells (Figure 3H), and average fat droplets area (µm^2^) (Figure 3I) were significantly reduced in LPHs-treated mice in comparison to WD group.

### 3.4. LPHs Modulate Hepatic Lipid Metabolism-Related Genes

**Figure 4:**
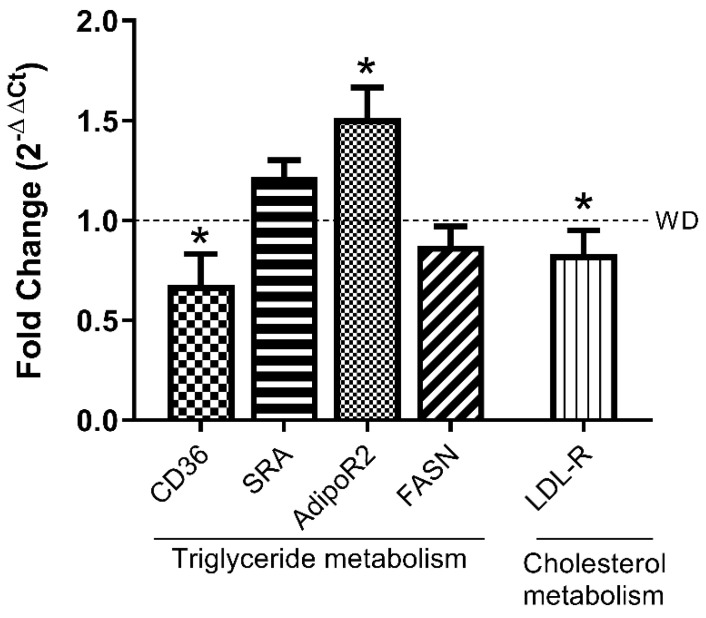
Lipid metabolism markers gene expression. Relative gene expression of scavenger receptors (CD36, SR-A), adiponectin receptor II (adipoR2), fatty acid synthase (FASN), and low density lipoprotein receptor (LDL-R) in the liver of mice after 12 week-treatment with LPHs. Data are shown as the mean of 2^−ΔΔCt^ and standard error of the mean of each group (*n*=10). * *p* ≤ 0.05, with respect to the WD group. CD36, cluster of differentiation 36; SR-A, class A macrophage scavenger receptor; WD, western diet group.

To evaluate whether LPHs could be affecting the expression of key genes involved in the hepatic lipid metabolism, RT-qPCRs were performed (Figure 4). The CD36 gene expression was significantly reduced in LPHs-treated mice in comparison to the WD group whereas no differences were observed in the SR-A gene expression. Moreover, a reduction in the LDL-R gene expression in the WD+LPHs group compared to WD group was observed. In addition, LPHs treatment significantly increased the hepatic mRNA relative expression of AdipoR2 and a trend towards a reduction was observed in mRNA of FASN. Interestingly, a significantly strong positive correlation between hTG-CD36 and hTC-LDL-R was shown (Table 1).

### 3.5. LPHs Increase the Hepatic Antioxidant Capacity

**Figure 5:**
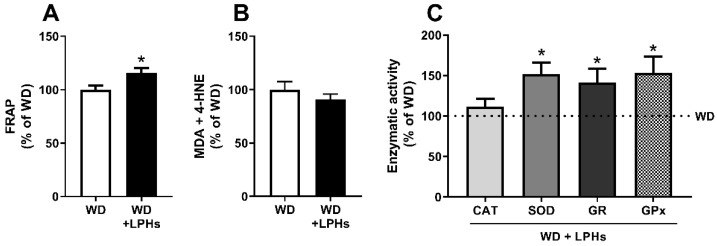
Hepatic antioxidant status. FRAP (**A**), MDA and 4-HNE levels (**B**) and CAT, SOD, GR, and GPx antioxidant activities (**C**) in the liver of ApoE^−/−^ mice after 12 weeks of treatment with LPHs. After referring the data to mg of protein, results are expressed as a percentage of the WD group. Data represent the mean and standard error of the mean of each group (*n* = 15). The pointed line represents WD group, while the bars represent the LPHs effects with respect to WD group (**C**). * *p* ≤ 0.05 with respect to the WD group. 4-HNE, 4-hydroxy-2-nonenal; CAT, catalase; FRAP, ferric reducing antioxidant power; GPx, glutathione peroxidase; GR, glutathione reductase; GSH, glutathione; MDA, malondialdehyde; SOD, superoxide dismutase; WD, western diet group; WD+LPHs, western diet + lupin protein hydrolysates group.

A significant increase of the hepatic FRAP was detected in the WD+LPHs group in respect to the WD group (Figure 5A). Furthermore, a trend towards a decrease in the lipid peroxidation products (MDA+4-HNE) was observed in LPHs-treated mice (Figure 5B). In fact, a significant negative correlation between hepatic FRAP and lipid peroxidation was detected in the LPHs-treatment group. (Table 1). A significant enhancement in the SOD, GPx and GR activities as well as a trend towards an increase in CAT activity were recorded in the LPHs-treated mice liver (Figure 5C) Accordingly, GPx and GR activities showed a strong positive correlation with FRAP (Table 1).

### 3.6. LPHs Improve the Liver Anti-Inflammatory Environment

**Figure 6:**
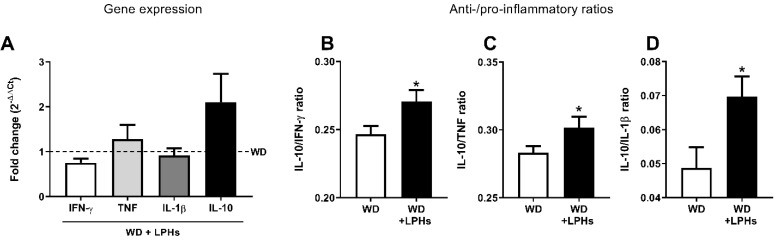
Liver inflammation. Relative gene expression of IFN-γ, TNF, IL-1β, and IL-10 in the liver of mice after 12 weeks of treatment with LPHs (**A**). The pointed line represents WD group, while the bars represent the LPHs effects with respect to WD group. Ratios between IL-10 and pro-inflammatory cytokines IFN-γ (**B**), TNF (**C**), IL-1β (**D**) expression (*n* = 10). Results are represented as the mean and standard error of the mean of each group. * *p* ≤ 0.05 with respect to the WD group. IFN-γ, interferon-γ; IL, interleukin; TNF, tumor necrosis factor; WD, western diet group; WD+LPHs, western diet + lupin protein hydrolysates group.

Although the hepatic gene expression of pro-inflammatory cytokines TNF, IL-1β, IFN-γ, and anti-inflammatory cytokine IL-10 did not show individual differences between the groups (Figure 6), a significant increase in the ratios of IL-10/IFN-γ, IL-10/IL-1β, and IL-10/TNF were observed (Figure 6). Moreover, a strong significant positive correlation was observed between IL-10/TNF ratio and FRAP (Table 1).

## 4. Discussion

This study describes the beneficial effects of a 12 week-treatment of LPHs on WD-induced abdominal adiposity and MAFLD in ApoE^−/−^ mice. LPHs reduced WAT size, hepatic lipid accumulation and liver oxidative stress and promoted a hepatic anti-inflammatory environment, critical components for the triggering and development of MAFLD. The effects of LPHs were related to neither the diet intake nor the BWG of mice that remained unchanged in the two experimental groups (WD and WD + LPHs).

MAFLD is the most common liver disorder worldwide [2,5]. Its incidence has been increasing over the last years, now affecting up to 25% of the general population [32]. Due to the strong socioeconomic and sanitary global impact caused by MAFLD [33], the interest in potential treatments has increased in the last decade [34]. ApoE^−/−^ mice is the principal genetic murine model of MAFLD [34]. In this regard, recent studies have shown steatosis, inflammation, and hepatic collagen accumulation in ApoE^−/−^ mice fed with high fat diet (HFD) [26].

The principal risk factor of MAFLD is the presence of metabolic syndrome of which the abdominal obesity is the primary component [35]. In fact, 80–90% of obese adults present MAFLD [36]. In this study, we observed that LPHs-treated mice not only presented a smaller size of abdominal WAT, but also a reduction of the adipocyte hypertrophy. As far as we know, this is the first study to report the beneficial effect of LPHs on abdominal obesity.

Hepatic steatosis, consisting in the accumulation of more than 5% lipids in hepatocytes, is the initial stage of MAFLD [37]. Our results showed that LPHs treatment reduced hTC and hTG in WD-fed ApoE^−/−^ mice. Furthermore, the histological liver analysis confirmed a LPHs-induced reduction of hepatic steatosis. Previous studies have demonstrated that both whole protein and lupin protein isolates (from *L. albus* and *L. luteus*) decrease hepatic lipids in rats [38,39], chicks [40] and hamsters [41], supporting our results. Furthermore, lactating rats fed with lupin whole protein (from *L. angustifolius*) show lower concentrations of hTC [42]. Wistar albino rats treated with LPHs (from *L. albus*) show less concentration of hTG in comparison to the Ctrl group [43]. Also, an octapeptide isolated from *L. angustifolius*, reduced hTG, and hTC in HFD-fed mice [44]. Despite the hepatic lipid-lowering effect of lupin has been demonstrated, none of the previous studies have evaluated neither the effect of LPHs from *L. angustifolius* nor the combined effects in the three most critical stages of MAFLD (fat accumulation, oxidative stress and inflammation) in mice fed with WD (a nutrition model more similar to that followed by a large part of the population in developed countries). In accordance with these results, we have recently demonstrated the plasmatic-cholesterol lowering effect of LPHs in ApoE^−/−^ mice (Santos et al., under review).

In order to analyse the underlying mechanisms of the LPHs effect on the liver fat accumulation, the mRNA expression of key genes involved in the liver lipid metabolism was quantified. We observed a significant reduction in the CD36 hepatic gene expression in LPHs-treated mice. The transmembrane glycoprotein CD36 plays a fundamental role in the steatosis process providing the hepatic long-chain fatty acids uptake [45]. Thus, CD36 contributes to the hepatic fat accumulation and subsequent metabolic dysfunctions [46,47,48]. Previous studies have demonstrated the pathogenic role of CD36 in rodents fed with HFD [49] and a significant upregulation of CD36 associated with an increased steatosis in patients with NASH [13]. To the best of our knowledge, there are no previous studies showing a reduction of hepatic CD36 expression caused by a vegetable hydrolysate.

In this regard, we suggest that the reduced levels of hTG and hepatic steatosis observed in LPHs-treated mice could be caused by a lower fatty acids uptake, which is induced by the downregulation of hepatic CD36. In accordance, correlation analyses showed a significant strong positive correlation between CD36 expression and hTG levels.

Furthermore, the LDL-R expression was reduced in LPHs-treated mice. Previous reports have suggested that LDL-R expression is increased in the liver of HFD-fed mice as a compensatory response for the hepatic clearance of circulating LDL-cholesterol [50]. The reduction in the LDL-R expression observed in LPHs-treated mice may be related to the presence of lower levels of liver cholesterol. This connection is in line with the positive correlation detected between LDL-R and hepatic cholesterol levels.

We also observed an upregulation in the hepatic gene expression of AdipoR2, which facilitates the uptake of adiponectin into the liver, in LPHs-treated mice. The overexpression of hepatic AdipoR2 in C57BL/6 mice improves MAFLD in all stages whereas the inhibition of AdipoR2 aggravated the disease [51]. Furthermore, NASH patients present lower levels of hepatic adiponectin and AdipoR2 than healthy subjects [52]. Therefore, we suggest that the overexpression of hepatic AdipoR2 is, at least partly, responsible for the hepatic lipid-lowering effect observed in LPHs-treated mice. On the other hand, no significant differences in FASN gene expression were observed between groups. Therefore, we suggest that the lower fat content in the liver of LPHs-treated mice is not due to a reduction of de novo synthesis of triglycerides. According to the “multiple-hit” hypothesis, oxidative stress plays a fundamental role in the pathogenesis and development of MAFLD [8]. ROS generated as a consequence of hepatic fat accumulation cause lipid peroxidation and cytokine production, promoting the progression of simple steatosis to NASH [53]. In addition, ROS can affect the activity of several enzymes involved in lipid metabolism (β-oxidation and lipogenesis), perpetuating the liver injury. In this regard, clinical evidences have demonstrated the beneficial action of antioxidants in lipid metabolism dysfunction such as MAFLD. Both classical antioxidants (vitamins C and E) and other natural dietary antioxidants such as coffee components or flavonoids (hesperetin and derivates) have been described as potential therapies in MAFLD [54,55]. MAFLD patients have high levels of ROS and lipid peroxidation products, as well as decreased activity of the main antioxidant enzymes, compared to healthy subjects [56,57]. Furthermore, studies in humans and animals have shown a strong association between oxidative stress and the severity of MAFLD [58,59]. In this study, mice treated with LPHs presented higher SOD, GPx, and GR activities, and lower hepatic levels of lipid peroxidation products (MDA, and 4-HNE). These observations are of special interest given that the activity of main antioxidant enzymes decreases as the disease worsens [56,59]. Accordingly, we observed an increase in hepatic levels of FRAP in LPHs-treated mice. FRAP is a widely used assay to measure the “general antioxidant power”, based on the ability of a sample to reduce iron [60]. Previous studies have demonstrated that FRAP is decreased in steatosis patients compared to healthy patients. In fact, FRAP levels decline as the disease progresses to NASH [56]. To the best of our knowledge, this is the first time that LPHs have been shown to improve liver antioxidant status. Thus, LPHs, in addition to reducing hepatic lipids, could ameliorate the MAFLD progression to NASH, through reducing oxidative stress. The inverse correlations observed between FRAP and levels of hTG and lipidic peroxidation products support the close association between fat accumulation and an increased oxidative stress.

Besides oxidative stress, inflammation plays an important role in the transition from liver steatosis to NASH. Increased levels of hepatic lipids and the subsequent oxidative stress lead to pro-inflammatory cytokines production that exacerbates liver injury [8]. Some studies have shown that patients with MAFLD present elevated concentrations of IL-1β, IL-6, and TNF [61,62,63] and low concentrations of IL-10 [64]. Moreover, TNF and IL-10 levels have been shown to correlate with the disease stage [64,65]. In fact, IL-10^−/−^ mice fed with an HFD present more hTG content in comparison to the wild type group [66], while IFNα receptor 1 or TNFα receptor 1 knockouts mice fed with hyperlipidemic diets show an attenuated hepatic steatosis [67,68]. Also, the inhibition of IL-1β in hypercholesterolemic mice inhibited the progression of simple steatosis to NASH and liver fibrosis [69]. In the present study, we found that LPHs improve the hepatic anti-inflammatory environment through increasing hepatic IL-10/TNF, IL-10/IL-1β, and IL-10/IFN-γ ratios. Other protein hydrolysates from casein or potato have shown anti-inflammatory effects in the liver [70,71]. Furthermore, a synthetic peptide based on *L. angustifolius* (GPETAFLR) has been shown to possess anti-inflammatory effects in the liver of high-fat diet induced obese mice [44]. In this study, we demonstrated for the first time the hepatic anti-inflammatory role of LPHs in ApoE^−/−^ mice fed a WD, a preclinical model of MAFLD. The strong correlation observed between FRAP and IL10/TNF ratio in LPHs-treated mice re-enforces the interplay between the oxidative stress and inflammation in the context of fatty liver disease.

## 5. Conclusions

This study is the first to report the beneficial pleiotropic actions of LPHs on key steps of MAFLD pathophysiology, including abdominal adiposity and hepatic steatosis, oxidative stress and inflammation. Therefore, these results support the development of nutritional strategies based on LPHs to prevent MAFLD.

## Figures and Tables

**Table 1:** Nonparametric correlations.

Spearman’s Correlations	Parameters	r	*p*-Value
**Lipid markers**	CD36 and hTG	0.750 *	0.020
	LDL-R and hTC	0.821 *	0.034
	hTG and hTC	0.950 ***	0.000
	FRAP and hTG	−0.600	0.088
**Antioxidant and anti-inflammatory**	FRAP and IL10/TNF ratio	0.762 *	0.028
**Antioxidant activities**	FRAP and GR activity	0.573	0.066
	FRAP and GPx activity	0.641 *	0.025
	FRAP and SOD activity	0.478	0.166
	FRAP and [MDA + 4-HNE]	−0.786 *	0.027

Non-parametric Spearman’s correlations between lipid, antioxidant, and inflammatory parameters in WD+LPHs group (*n* = 10). * *p* ≤ 0.05, *** *p* ≤ 0.001. 4-HNE, 4-hydroxy-2-nonenal; CD36, cluster of differentiation 36; FRAP, ferring reduction antioxidant power; GPx, glutathione peroxidase; GR, glutathione reductase; hTC, hepatic total cholesterol; hTG, hepatic triglyceride; LDL-R, low density lipoprotein receptor; MDA, malondialdehyde.

## Data Availability

Data is contained within the article and Supplementary Materials.

## Supplementary Materials

The following are available online at https://www.mdpi.com/article/10.3390/antiox10081222/s1, Table S1. Composition of experimental Western diet; Table S2. Primers sequences and qPCR annealing temperature; Figure S1. Body weight parameters and daily food intake.
