# Supplementary material for: Lupinus angustifolius Protein Hydrolysates Reduce Abdominal Adiposity and Ameliorate Metabolic Associated Fatty Liver Disease (MAFLD) in Western Diet Fed-ApoE−/− Mice
Source: Antioxidants (Basel). 2021 Jul 29;10(8):1222. doi: 10.3390/antiox10081222 (PMC8388992; doi:10.3390/antiox10081222)
Supplement: Supplementary file 1 [file antioxidants-10-01222-s001.zip › antioxidants-1309251-supplementary.pdf]

antioxidants

MDPI

Supplementary Materials

Lupinus angustifolius protein hydrolysates reduce abdominal adiposity and ameliorate metabolic associated fatty liver disease (MAFLD) in Western diet fed-ApoE$^{-/-}$ mice.

Guillermo Santos-Sánchez[1,2], Ivan Cruz-Chamorro[1,2*], Ana Isabel Álvarez-Ríos[3], José María Fernández-Santos[4], María Victoria Vázquez-Román[4], Beatriz Rodríguez-Ortiz[1,+], Nuria Álvarez-Sánchez[1,+], Ana Isabel Álvarez-López[1,2], María del Carmen Millán-Linares[5], Francisco Millán[6], Justo Pedroche[6], María Soledad Fernández-Pachón[7], Patricia Judith Lardone[1,2], Juan Miguel Guerrero[1,2,3], Ignacio Bejarano[1,2], Antonio Carrillo-Vico[1,2*]

Table 1S. Composition of experimental Western diet.

Ingredients	%
**Fat (2.12 kcal/g)**	
Lard	20.68
Soybean oil	2.91
**Carbohydrate (1.65 kcal/g)**	
Sucrose	20.09
Maltodextrin	11.65
Dextrin	8.48
Powdered cellulose	5.82
**Protein (0.83 kcal/g)**	
Casein- vitamin tested	23.30
**Vitamins**	1.16
**Minerals**	1.16
**Food additives**	
**Potassium citrate and tribasic monohydrate**	1.92
Calcium phosphate	1.51
Calcium carbonate	0.64
L-Cystine	0.35
Choline bitartrate	0.23
Food colouring (FD&C Red No.40)	0.05
**Total energy (4.60 Kcal/g)**	
Fats (ether extract)	46.1
Carbohydrates	35.8
Proteins	18.1

Ingredients percentage of Western diet and energy provided from the different macromolecules.

antioxidants

MDPI

Figure S1. Body weight parameters and daily food intake.

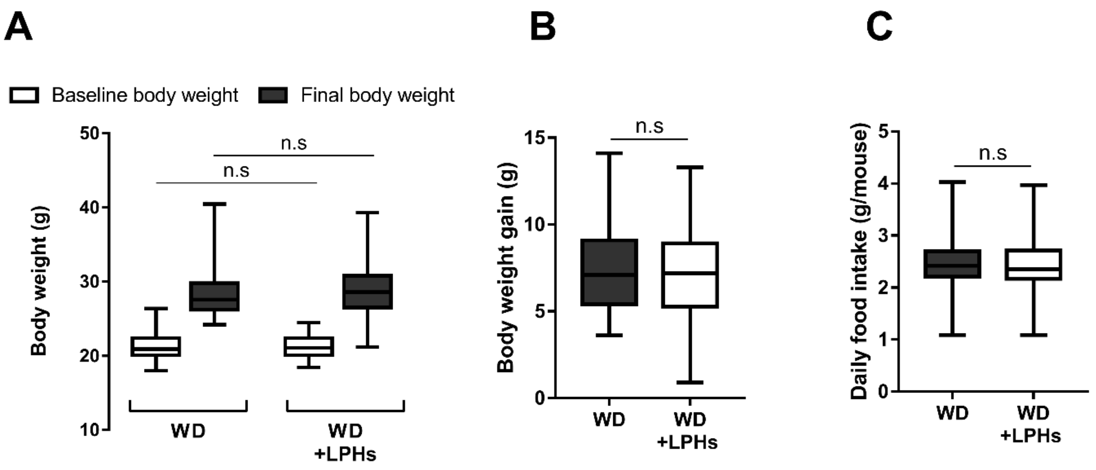

Baseline body weight and final body weight (A), body weight gain (B), and daily food intake (C) of both experimental groups. Values are shown as the mean and standard error of the mean of each group (n=30). n.s., non significant; WD, western diet group; WD+LPHs, western diet + lupine protein hydrolysates group.
